# Dietary Magnesium Intake Modifies the Association Between Vitamin D and Systolic Blood Pressure: Results From NHANES 2007–2014

**DOI:** 10.3389/fnut.2022.829857

**Published:** 2022-02-24

**Authors:** Weichao Huang, Xiaoman Ma, Yue Chen, Jiayi Zheng, Haojia Li, Ayinigaer Nizhamu, Qingting Hong, Xuguang Guo

**Affiliations:** ^1^Department of Clinical Laboratory Medicine, The Third Affiliated Hospital of Guangzhou Medical University, Guangzhou, China; ^2^Department of Clinical Medicine, The Second Clinical School of Guangzhou Medical University, Guangzhou, China; ^3^Department of Clinical Medicine, The Third Clinical School of Guangzhou Medical University, Guangzhou, China; ^4^Department of Clinical Laboratory Medicine, Key Laboratory for Major Obstetric Diseases of Guangdong Province, The Third Affiliated Hospital of Guangzhou Medical University, Guangzhou, China; ^5^Department of Clinical Laboratory Medicine, Key Laboratory of Reproduction and Genetics of Guangdong Higher Education Institutes, The Third Affiliated Hospital of Guangzhou Medical University, Guangzhou, China

**Keywords:** vitamin D, dietary magnesium intake, systolic blood pressure (SBP), interaction, metabolism

## Abstract

**Introduction:**

Although the association between blood pressure and vitamin D has been well studied, the effects of dietary magnesium intake on this relationship are still unclear. Thus, this study aimed to determine the effects of dietary magnesium intake on the association between vitamin D and blood pressure.

**Methods:**

The present study analyzed data from the continuous the National Health and Nutrition Examination Survey (NHANES) 2007–2014. We included 8,799 participants aged 20 years or older. Multivariable linear regression was performed to assess the association between vitamin D and systolic blood pressure (SBP) and diastolic blood pressure (DBP). Dietary magnesium intake was stratified by low magnesium intake (<299 mg/d) and high magnesium intake (≥299 mg/d). Effect modification by dietary magnesium intake was assessed through interaction tests between vitamin D and SBP in the multivariable linear regression.

**Results:**

In this cross-sectional study, we found that vitamin D was negatively related to SBP, but not to DBP. The relationship between vitamin D and SBP was different in the low and high magnesium intake group (β: −0.25 95%Cl: −0.4~0.07 vs β: −0.32 95%Cl: −0.52~-0.12). Furthermore, magnesium intake significantly modified the negative relationship between vitamin D and SBP in most of the models.

**Conclusion:**

Our research showed that magnesium and vitamin D have an interactive effect in reducing SBP, which may have great importance for clinical medication.

## Introduction

Hypertension is a global public health problem with the prevalence of nearly 40% in adults over 25 years of age worldwide (1). It can be the risk factor of some cardiovascular diseases, including stroke and heart failure (2). However, the underlying mechanism of hypertension is not clear, and it cannot be cured so far (3).

Vitamin D deficiency (VDD) is highly prevalent worldwide (4). It is associated with pre-eclampsia, childhood dental caries, periodontitis, cardiovascular diseases, and so on (5). Recent studies have pointed out the relationship between vitamin D and blood pressure (BP) (6, 7). Observational studies in Meta-analysis have also shown that VDD is associated with higher BP (8). Studies in animals and humans suggested that VDD can activate the renin-angiotensin system (RAS), which promotes the development of hypertension (9). In addition, Sakamoto R found that 25(OH)D levels were negatively correlated with systolic blood pressure, but the relationship between serum 25(OH)D and diastolic blood pressure was non-significant (10). However, a prospective cohort study by Myriam Abboud showed no association between vitamin D and BP (11). The differences in the results of the studies may be attributed to potential effect modifiers or confounding factors that have not been fully considered, such as dietary magnesium intake.

Previous studies have shown that the enzymes that synthesize and metabolize vitamin D depend on magnesium (12). Recent observational studies have shown that magnesium and vitamin D have a significant interaction, and the inverse association between serum vitamin D and risk of mortality due to cardiovascular disease and colorectal cancer is present only when the intake of magnesium is above the median (13). However, limited clinical studies have assessed the effect of magnesium intake on vitamin D and BP (12, 14). Therefore, we hypothesized that magnesium interacts with vitamin D on BP. This cross-sectional study aims to explore the association between serum vitamin D and BP and the modifying effect of magnesium intake on this association.

## Methods

### Data Source

Our stages of the National Health and Nutrition Examination Survey (NHANES) 2007–2008, 2009–2010, 2011–2012, and 2013–2014 were used in the present study. NHANES is a health-related program that includes a nationally representative cross-sectional survey of the non-institutionalized civilian population of the United States. Demographic, socioeconomic, and health-related information was obtained through questionnaires and physical and laboratory examinations. Health interviews were conducted at the participants' homes, while extensive physical examinations, including blood sample collection, were conducted at the Mobile Inspection Center (MEC). The serum specimens were then tested at the Division of Laboratory Sciences. Before participating, all participants provided written informed consent, and the study was approved by the NCHS Research Ethics Review Board (https://wwwn.cdc.gov/nchs/nhanes/default.aspx).

### Measurement of Vitamin D Status

The laboratory specimens for the measurement of 25(OH)D status collected during the MEC examination were centrifuged, aliquoted, and transported in cold storage to the Center for Disease Control (CDC) Environment Health Laboratory, where 25-hydroxyvitamin D3[25(OH)D3], 25-hydroxyvitamin D2[25(OH)D2], and 3-epi-25-hydroxyvitamin D3[3-epi-25(OH)D3] concentrations were examined using the ultra-high-performance liquid chromatography-tandem mass spectrometric method (UHPLC-MS/MS). Serum 25(OH)D3 and 25(OH)D2, the major circulating forms of vitamin D, were summed and defined as total serum 25(OH)D.

### Magnesium Intake

Dietary data regarding magnesium intake was obtained *via* a precise list of all foods consumed by an individual during the former period of 24 h. The 24-h recall method is most often used for determining dietary intake in large-scale surveys. The decision to continue with this method over the years in NHANES has been based on a consensus of expert groups during workshops held periodically to evaluate data collection methods in NHANES. The previous 24 h' dietary information this study used was from a 24-h dietary recall interview collected in-person in the MEC. The daily magnesium intake was defined based on the average value of the overall population as high (>299 mg/d) or low intake ( ≤ 299 mg/d).

### Blood Pressure Measurement

Blood pressure (BP), the main outcome variable, was measured with a mercury sphygmomanometer by trained staff according to standardized protocols (15) with the participant in a seated position. The systolic blood pressure (SBP) and diastolic blood pressure (DBP) are respectively defined as the point where the first Korotkoff sound is heard and the mercury level 2 mm below the point where the last sound is heard. In the present study, we calculated the average of up to 3 brachial systolic and diastolic BP readings for further analyses.

### Covariates

Since several factors may affect the outcomes, the participants' age, gender, race/ethnicity, the season of examination, physical activity, educational level, alcohol consumption, smoking status, calcium intake, body mass index (BMI), family income, and biochemical indexes including triglyceride, cholesterol, and HDL-cholesterol were selected as the potential covariates in our analysis models. Race/ethnicity was categorized as Mexican American, other Hispanic, Non-Hispanic white, Non-Hispanic black, and other races. Educational level was categorized as less than high school, high school graduation, and college or above. According to the time of the NHANES survey, the season of examination was classified as winter months (November to April) or summer months (May to October). Data on alcohol drinking (yes = at least 12 alcohol drinks per year vs. no = <12 alcohol drinks per year) was obtained by questionnaire interviews. Smoking status is divided into current smokers (who have smoked more than 100 cigarettes in a lifetime and currently smoke), former smokers (who have smoked more than 100 cigarettes in a lifetime but have not smoked), and non-smokers (who have never smoked more than 100 cigarettes). Physical activity is defined as vigorous work activity, moderate work activity, walk or bicycle, vigorous recreational activities, and moderate recreational activities according to the level of activity intensity. Respondents who answered yes to the following questions were classified as being diagnosed with high blood pressure: “Have you ever been told by a doctor or other health professional that you had hypertension or so-called high blood pressure?” To assess family income, we selected the poverty income ratio (PIR), which was calculated by the family size-specific threshold. PIR was categorized as <1 (below the poverty line), 1–3, and >3. Moreover, the specific information concerning serum contents of triglyceride, cholesterol, HDL-cholesterol was extracted from the NHANES laboratory detection data.

### Statistical Analysis

All the analyses were conducted using the statistical software packages R (http://www.R-project.org, The R Foundation) and Free Statistics software version 1.3 (16). The complex multistage stratified sampling design of NHANES was illustrated by the use of appropriate strata, clusters, and weights in the statistical analysis process. To examine the association between vitamin D and BP, multivariate linear regression procedures were performed. SBP and DBP means were respectively evaluated across strata of magnesium intake. Interaction among subgroups was inspected by the likelihood ratio test.

Additionally, 95% Cls were calculated. The level of statistical significance was set at *p* < 0.05. Continuous variables are expressed as mean and SD or median and interquartile range (IQR), and categorical variables are expressed as weighted percentages (%) in descriptive analysis. The Chi-square tests (categorical variables) and the *t*-test (normal distribution), and the Kruskal-Wallis (skewed distribution) test are respectively performed to evaluate continuous variables and categorical variables.

## Results

### Baseline Characteristics of the Study Participants

**Figure 1:**
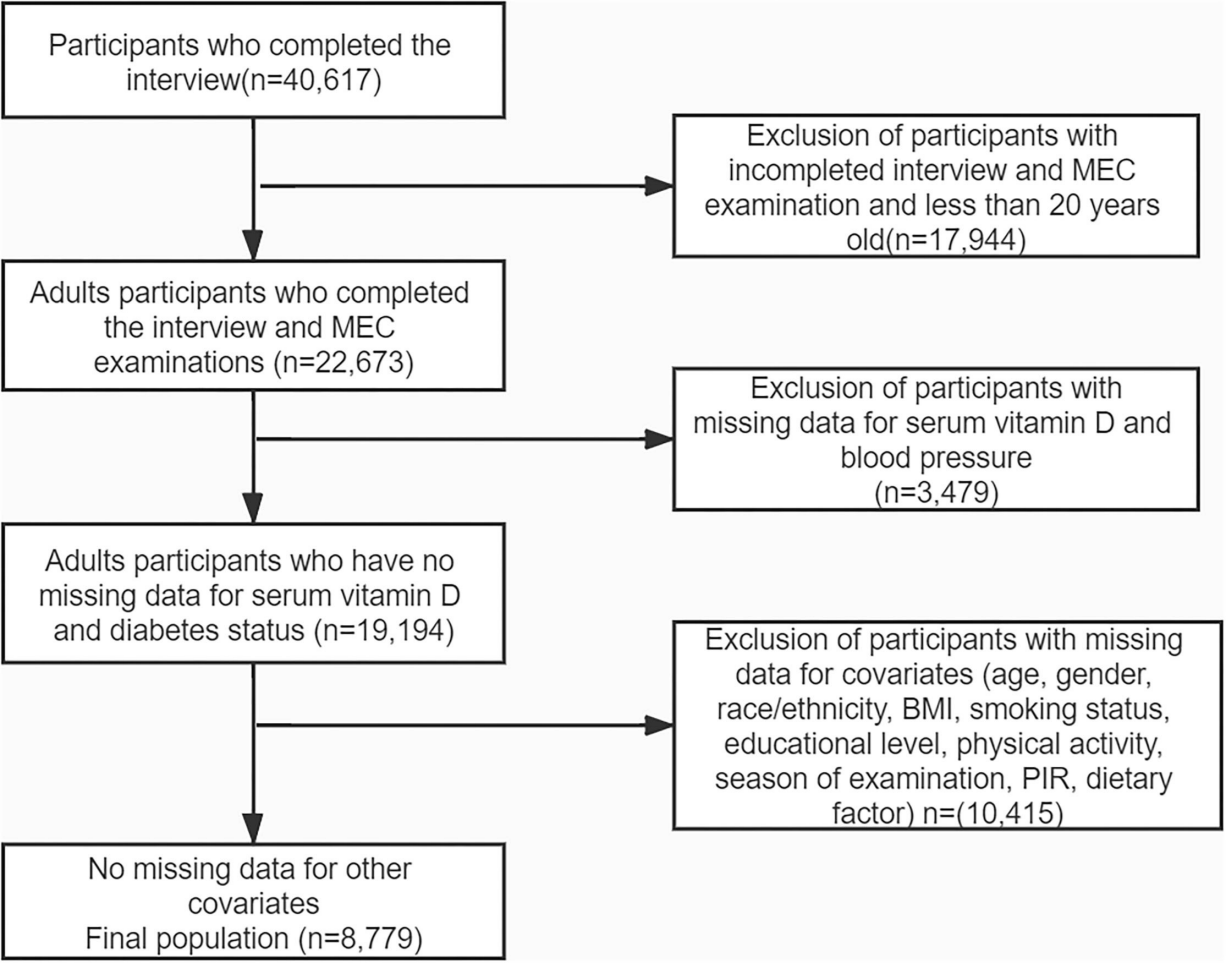
Flowchart of participants enrollment.

This study used four cycles of NHANES 2007–2008, 2009–2010, 2011–2012, and 2013–2014. We enrolled 40,617 participants, 22,673 adults (≥20 years old) who completed the interview, and MEC examination was enrolled in our study. Participants with missing data on serum 25-hydroxyvitamin D concentration (*n* = 2,018) and blood pressure (*n* = 2,257) were excluded. After excluding participants with missing data for covariates, our analysis included 8,779 participants in total. The flowchart of the exclusion criteria is summarized in Figure 1. The descriptive characteristics of participants were displayed in Table 1 based on dietary magnesium intake. Compared with the low magnesium intake (<299 mg/d), participants with high magnesium intake (≥299 mg/d) were more likely to be younger, male, non-Hispanic white, had lower BMI, received a good education, PIR> 3, higher intake of alcohol, dietary vitamin D, dietary calcium, dietary magnesium, dietary energy intake, and higher value of triglycerides. Furthermore, individuals with high magnesium intake had a low prevalence of hypertension. No statistically significant differences were detected in the season of examination, smoking status, physical activity, cholesterol, and direct HDL-cholesterol (all *p* > 0.05).

**Table 1:** Characteristics of participants.

**Variables**	**Dietary magnesium intake (mg/d)**			
	**Total (*n* = 8,779)**	** <299 mg/d (*n* = 5,077)**	**≥299 mg/d (*n* = 3,702)**	***p*-value**
Age (years), Mean ± SD	49.1 ± 17.7	50.0 ± 18.3	47.8 ± 16.8	<0.001
Gender,n(%)				<0.001
Male	4,413 (50.3)	2,095 (41.3)	2,318 (62.6)	
Female	4,366 (49.7)	2,982 (58.7)	1,384 (37.4)	
Race/Ethnicity, *n* (%)				<0.001
Mexican America	1,253 (14.3)	647 (12.7)	606 (16.4)	
Other Hispanic	862 (9.8)	509 (10)	353 (9.5)	
Non-Hispanic white	4,145 (47.2)	2,337 (46)	1,808 (48.8)	
Non-Hispanic black	1,741 (19.8)	1,161 (22.9)	580 (15.7)	
Other races	778 (8.9)	423 (8.3)	355 (9.6)	
Season of examination, n (%)				0.563
Winter	4,046 (46.1)	2,326 (45.8)	1,720 (46.5)	
Summer	4,733 (53.9)	2,751 (54.2)	1,982 (53.5)	
BMI(kg/m**2), Mean ± SD	29.0 ± 6.6	29.3 ± 6.8	28.6 ± 6.4	<0.001
Education level, *n* (%)				<0.001
Did not graduate from high school	2,155 (24.6)	1,398 (27.6)	757 (20.5)	
Graduated from high school	1,974 (22.5)	1,246 (24.6)	728 (19.7)	
College education or above	4,644 (52.9)	2,428 (47.9)	2,216 (59.9)	
PIR, *n* (%)				<0.001
<1	1,847 (21.0)	1,183 (23.3)	664 (17.9)	
1–3	3,651 (41.6)	2,232 (44)	1,419 (38.3)	
>3	3,281 (37.4)	1,662 (32.7)	1,619 (43.7)	
Smoking status, n (%)				0.354
Current smoker	1,883 (21.4)	1,112 (21.9)	771 (20.8)	
Former smoker	2,098 (23.9)	1,221 (24)	877 (23.7)	
Never smoker	4,798 (54.7)	2,744 (54)	2,054 (55.5)	
Physical activity, *n* (%)				0.489
Vigorous work activity	1,575 (17.9)	910 (17.9)	665 (18)	
Moderate work activity	1,829 (20.8)	1,051 (20.7)	778 (21)	
Walk or bicycle	1,249 (14.2)	717 (14.1)	532 (14.4)	
Vigorous recreational activities	596 (6.8)	327 (6.4)	269 (7.3)	
Moderate recreational activities	3,530 (40.2)	2,072 (40.8)	1,458 (39.4)	
Had at least 12 alcohol drinks/lifetime, n (%)				<0.001
Yes	6,478 (73.8)	3,533 (69.6)	2,945 (79.6)	
No	2,291 (26.1)	1,537 (30.3)	754 (20.4)	
Don't know	10 (0.1)	7 (0.1)	3 (0.1)	
Be diagnosed with high blood pressure, *n*(%)				<0.001
Yes	3,140 (35.8)	1,925 (37.9)	1,215 (32.8)	
No	5,626 (64.1)	3,145 (61.9)	2,481 (67)	
Don't know	13 (0.1)	7 (0.1)	6 (0.2)	
Dietary vitamin D (D2 + D3) intake(mcg),Median (IQR)	3.1 (1.2, 6.0)	2.2 (0.8, 4.4)	4.9 (2.3, 8.3)	<0.001
Dietary calcium intake (mg),Median (IQR)	815.0 (535.0, 1,188.0)	631.0 (421.0, 892.0)	1,128.0 (823.0, 1,552.0)	<0.001
Dietary magnesium intake(mg),Median (IQR)	270.0 (195.0, 367.0)	207.0 (161.0, 249.0)	389.0 (336.2, 480.0)	<0.001
Dietary energy intake(kcal),Median(IQR)	1,950.0 (1,441.0, 2,612.0)	1,593.0 (1,223.0, 2,043.0)	2,578.0 (2,032.0, 3,285.0)	<0.001
Cholesterol (mmol/L), Mean ± SD	5.0 ± 1.1	5.0 ± 1.1	5.0 ± 1.1	0.664
Triglycerides (mmol/L), Mean ± SD	1.4 (0.9, 2.1)	1.4 (0.9, 2.1)	1.4 (0.9, 2.2)	0.003
Direct HDL-Cholesterol (mmol/L), Mean ± SD	1.4 ± 0.4	1.4 ± 0.4	1.4 ± 0.4	0.401

*PIR: poverty income ratio; BMI: body mass index*.

### Association Between Serum Vitamin D and BP

As shown in Table 2, vitamin D was negatively associated with DBP in the unadjusted model and model2. But in turn, after the adjustment of the confounding factors in the fully adjusted model, vitamin D was not associated with DBP(*p* > 0.05). In the fully adjusted model, vitamin D was negatively associated with SBP (β: −0.28, Cl: −0.41, −0.14).

**Table 2:** Association between serum vitamin D and blood pressure.

**Models**	**n**	**DBP**		**SBP**	
		**β_95Cl**	***P*_value**	**β_95Cl**	***P*_value**
Model 1	8,779	−0.23 (−0.33~-0.14)	<0.001	0 (−0.14~0.13)	0.976
Model 2	8,779	−0.12 (−0.22~-0.02)	0.024	−0.36 (−0.49~-0.23)	<0.001
Model 3	8,779	−0.05 (−0.15~0.06)	0.401	−0.21 (−0.34~-0.07)	0.002
Model 4	8,779	−0.05 (−0.16~0.05)	0.328	−0.19 (−0.33~-0.06)	0.005
Model 5	8,779	−0.06 (−0.17~0.05)	0.263	−0.22 (−0.35~-0.08)	0.002
Model 6	8,779	−0.05 (−0.15~0.06)	0.395	−0.23 (−0.37~-0.1)	0.001
Model 7	8,779	−0.06 (−0.17~0.04)	0.247	−0.28 (−0.41~-0.14)	<0.001

*Model 1: not adjusted; Model 2: adjusted for age, sex, race/ethnicity; Model 3: model2+BMI,PIR,education level, smoking status, physical activity, alcohol use, season of examination; Model 4: model3+dietary magnesium intake, dietary calcium intake, dietary vitamin D intake; Model 5: model4+cholesterol,triglycerides, HDL-Cholesterol; Model 6: model 5+dietary energy intake; Model 7: model 6+be diagnosed with high blood pressure DBP: diastolic blood pressure; SBP:systolic blood pressure; PIR: poverty income ratio; BMI: body mass index*.

### Magnesium Intake Affects the Association Between Vitamin D and SBP

Table 3 shows that in model1~6, the association between vitamin D and SBP was significant in the high magnesium intake group (≥299 mg/d), but not stably in the low magnesium group (<299 mg/d). In the fully adjusted model, the high magnesium intake group, the beta-value for the high magnesium intake group was still lower than the low magnesium intake group, although the interaction effect was statistically non-significant (*p* for interaction = 0.111). However, the association between vitamin D level and DBP was not significant in the high nor the low magnesium intake group (Supplementary Table 1).

**Table 3:** Interactive effect of vitamin D and dietary magnesium intake on systolic blood pressure (SBP).

**Models**	**Low-magnesium intake** **(<299 mg/d**, ***n*** **=** **5,077)**		**High-magnesium intake (≥299 mg/d**, ***n*** **=** **3,702)**		***p* for interaction**
	**β(95%Cl)**	***P*-value**	**β (95%Cl)**	***P*-value**	
Model 1	0.18 (0~0.36)	0.044	−0.24 (−0.45~-0.04)	0.021	0.003
Model 2	−0.28 (−0.45~-0.1)	0.002	−0.46 (−0.67~-0.26)	<0.001	0.017
Model 3	−0.15 (−0.32~0.03)	0.102	−0.29 (−0.5~-0.08)	0.006	0.02
Model 4	−0.15 (−0.33~0.03)	0.093	−0.28 (−0.49~-0.07)	0.008	0.024
Model 5	−0.18 (−0.35~0)	0.052	−0.3 (−0.51~-0.1)	0.004	0.026
Model 6	−0.19 (−0.36~-0.01)	0.039	−0.3 (−0.51~-0.1)	0.004	0.035
Model 7	−0.25 (−0.42~-0.07)	0.006	−0.32 (−0.52~-0.12)	0.002	0.111

*Model 1: not adjusted; Model 2: adjusted for age, sex, race/ethnicity; Model 3: model2+BMI,PIR,education level, smoking status, physical activity, alcohol use, season of examination; Model 4: model3+ dietary calcium intake, dietary vitamin D intake; Model 5: model4+cholesterol,triglycerides, HDL-Cholesterol; Model 6: model 5+dietary energy intake; Model 7: model 6+be diagnosed with high blood pressure DBP: diastolic blood pressure; SBP: systolic blood pressure; PIR: poverty income ratio; BMI: body mass index*.

## Discussion

Analyzing the nationally representative adult population data in the United States, this study showed that vitamin D was negatively related to SBP, and has no significant relationship with DBP. Besides, it was found that dietary magnesium intake and vitamin D had an interactive effect on reducing SBP in most of the models, which indicates that the interaction of high serum vitamin D and high intake of magnesium is greater than the sum of the individual effects.

To the best of our knowledge, this is the first large-scale study to assess the interaction of dietary magnesium intake on the association of vitamin D and SBP. At present, many mechanisms for vitamin D to lower blood pressure have been proposed, but the exact mechanism is still unclear. One possible mechanism is that vitamin D can down-regulate the activity of the renin-angiotensin-aldosterone system (RASS) by inhibiting the production of renin and lowering BP (17). And some potential effects are that vitamin D can inhibit the production of parathyroid hormone, improve endothelial cell function, and reduce the production of pro-inflammatory factors (18–20). According to these possible mechanisms, the blood pressure levels can be directly or indirectly affected by vitamin D. Similar to our study, Karani S. Vimaleswaran (7) used a Mendelian randomization study to evaluate whether BP and hypertension risk can be modified by 25(OH) D concentration. Sheng Hui Wu also gave the same conclusion in his study (21). A meta-analysis suggested that vitamin D supplementation slightly reduced SBP by 1.964 mmHg, but did not reduce DBP, and the decrease in SBP was not dependent on the dose (22). Joukar F. conducted a prospective cohort study that found that the relationship between vitamin D levels and SBP was weak but statistically negative, and there was no significant relationship between vitamin D levels and DBP (23). However, a randomized trial showed that vitamin D treatment did not affect the blood pressure of the patients compared to placebo (24). A review (25) and a meta-analysis (26) revealed that vitamin D supplementation did not reduce blood pressure.

Epidemiological and clinical studies have shown that magnesium supplementation is negatively correlated with increased BP (27). Similar to vitamin D for lowering blood pressure, magnesium can down-regulate the activity of the RASS system by inhibiting the production of aldosterone (27). Wong NL agreed that dietary magnesium intake can promote the release of rat atrial natriuretic peptide (28), a kind of hormone that can antagonize RAAS by inhibiting the secretion of renin and the production of aldosterone (29). There are also some mechanisms explaining that magnesium can reduce vascular calcification (27) and inhibit the production of NO (30), thereby inhibiting the increase in BP. In a randomized double-blind controlled trial, magnesium supplementation can reduce SBP in pregnant women (31–33). Another randomized controlled trial proved that magnesium is negatively related to BP (34). Zorica Rasic-Milutinovic conducted a cross-sectional study that magnesium in drinking water helps prevent hypertension (35).

Magnesium is quite important for the metabolism of vitamin D in the human body. Vitamin D is metabolized into 1, 25 (OH)2D active form through liver 25-hydroxylation and kidney 1α-hydroxylation, which is a magnesium-dependent process (12, 36). And in this process, vitamin D needs to be combined with vitamin D-binding protein, and magnesium is a cofactor of vitamin D-binding protein. Therefore, high magnesium intake is conducive to the enhancement of vitamin D activity. This mechanism can explain the interaction effect found in the present study. A review suggests that magnesium supplementation may reduce the risk of vitamin D deficiency-related complications (36). Data shows that people with high magnesium intake have a lower risk of vitamin D deficiency or insufficiency (13). Dai et al. (12) claims that serum 25(OH) D will increase significantly only when supplemented with vitamin D and magnesium. For diseases treated with vitamin D, adequate magnesium supplementation should be considered at the same time, which requires further clinical trials to prove.

Some limitations exist in our research. First, we cannot prove causality or directionality because of the cross-sectional design. And the results might be confounded by some other unmeasured variables even after multiple adjustments. However, some potential confounding factors including some dietary factors were adjusted in the linear regression model. Second, there is no simple and accurate method to determine the total magnesium status of the human body (14, 27). We obtained the magnesium intake of participants through questionnaire surveys. Recall bias may occur because the dietary data comes from self-reported 24-h dietary recall. Third, serum vitamin D status was estimated from a single temporal measure. A marked circadian variation was demonstrated for 25(OH)D (37). We were not able to account for whether the circadian rhythm of serum vitamin D concentration has a possible impact on our results. Finally, although a large number of samples were included, the study population was limited to US residents. Therefore, consideration is necessary when inferring other populations. As a result, well-designed multi-center controlled trials are needed to verify our findings.

## Conclusion

In conclusion, our results indicate that vitamin D and SBP are negatively correlated. And this correlation was different in the high or low magnesium intake group. The interaction of magnesium on the association between vitamin D and BP may be of great significance to the clinical use of drugs for the prevention of hypertension.

## Data Availability Statement

The original contributions presented in the study are included in the article/Supplementary Material, further inquiries can be directed to the corresponding author.

## Ethics Statement

The survey protocol for NHANES has been approved by the Institutional Research Ethics Review Board of the CDC National Center for Health Statistics. All participants provided written informed consent, and the study was approved by the NCHS Research Ethics Review Board (https://wwwn.cdc.gov/nchs/nhanes/default.aspx). Human subjects were not involved in this study.

## Author Contributions

WH conducted the data collection and analysis. XM wrote the manuscript. WH and XM modified the manuscript. YC conducted the data interpretation. JZ drew the figure. HL conducted the data collection. AN made the table. XG designed the study and reviewed the manuscript. All authors contributed to the article and approved the submitted version.

## Conflict of Interest

The authors declare that the research was conducted in the absence of any commercial or financial relationships that could be construed as a potential conflict of interest.

## Publisher's Note

All claims expressed in this article are solely those of the authors and do not necessarily represent those of their affiliated organizations, or those of the publisher, the editors and the reviewers. Any product that may be evaluated in this article, or claim that may be made by its manufacturer, is not guaranteed or endorsed by the publisher.
