# Supplementary material for: Dietary Magnesium Intake Modifies the Association Between Vitamin D and Systolic Blood Pressure: Results From NHANES 2007–2014
Source: Front Nutr. 2022 Feb 24;9:829857. doi: 10.3389/fnut.2022.829857 (PMC8908235; doi:10.3389/fnut.2022.829857)
Supplement: Supplementary file 1 [file Table_1.pdf]

TABLE S1 Interactive effect of vitamin D and dietary magnesium intake on DBP

Models	low-magnesium intake (n=5077)		high-magnesium intake (n=3702)		p for interaction
	β(95%Cl)	P-value	β(95%Cl)	P-value	
Model 1	-0.24 (-0.37~-0.12)	<0.001	-0.26 (-0.41~-0.11)	0.001	0.86
Model 2	-0.12 (-0.26~0.01)	0.074	-0.13 (-0.3~0.04)	0.121	0.964
Model 3	-0.04 (-0.18~0.1)	0.555	-0.03 (-0.2~0.14)	0.71	0.732
Model 4	-0.04 (-0.18~0.1)	0.573	-0.04 (-0.21~0.14)	0.678	0.721
Model 5	-0.05 (-0.19~0.08)	0.448	-0.06 (-0.23~0.11)	0.508	0.748
Model 6	-0.05 (-0.18~0.09)	0.502	-0.04 (-0.21~0.12)	0.605	0.927
Model 7	-0.07 (-0.2~0.07)	0.339	-0.05 (-0.22~0.12)	0.547	0.868

Model 1: not adjusted;
Model 2: adjusted for age, sex, race/ethnicity;
Model 3: model2+BMI,PIR,education level, smoking status, physical activity, alcohol use, season of examination;
Model 4: model3+ dietary calcium intake, dietary vitamin D intake;
Model 5: model4+cholesterol,triglycerides, HDL-Cholesterol;
Model 6: model 5+dietary energy intake;
Model 7: model 6+be diagnosed with high blood pressure
DBP: diastolic blood pressure; SBP :systolic blood pressure; PIR: poverty income ratio; BMI: body mass index
